# ASO Author Reflections: The Right Care, for the Right Patient, at the Right Time: Development of the PREdiCT Screening Tool for Prehabilitation in Cancer Surgery

**DOI:** 10.1245/s10434-026-19466-8

**Published:** 2026-03-22

**Authors:** Jack Reeves, Cherry Koh, Michael Solomon, Kate Alexander, Daniel Steffens, Global (P)rehabilitation Initiative Collaborative Group, Sascha Karunaratne, Alexandria Petridis, Sharon Carey, Haryana M. Dhillon, Linda Denehy, Bernhard Riedel, Kate McBride, Chelsia Gillis, Kate White, Nicholas Hirst, Zaid Shadid, Ishraque Rahman, Neil Pillinger, Abdalla Hadhoud, Abdelatte Ghanam, Abdelilah Ghannam, Abdelmoiz Mohamed, Abdelrahim Ahmed, Abdelrahman Mohamedali, Abdirahman Osman, Abdulaleem A. Esmail, Abdulhamid Shaban, Abdulrahman Mohammed, Abdulzahra Hussain, Abdullah Al-Mehdhar, Abdullah Alyami, Achraf Drissi-El-Bouzaidi, Aeshah Alqathafi, Ahmed Ahmayda, Ahmed Elsayed Tharwat Abdelsalam, Ahmed Ibrahim MohamedMohamed, Ahmed Musbah, Ahmed Shoman, Ahmed Wahaballah, Aiman Al-Touny, Aiman Hussien, Akrem Amer, Amina Houmada, Amr Badawy, Andrea B. Maier, Andreas Denys, Andrew Davies, Angel Becerra-Bolaños, Angela Jerath, Anjum Khan Joad, Anthony Absalom, Areej Ahmed Al-Qaidani, Aras Emre Canda, Ashleigh Maske, Ashu Sara Mathai, Awab Hamid, Ayah Attallah, Ayten Saracoglu, Badr Aldeen Al-Tayar, Barbara Sinner, Basantha Kumar Rayani, Behic Girgin, Bernhard Riedel, Bisher Sawaf, Birgitte Brandstrup, Bodil Andersson, Brendan Moran, Brendon Roxburgh, Bruno Romanò, Carol Keen, Catarina Tiselius, Chelsia Gillis, Chloe Grimmett, Christian Beilstein, Cindy Yeoh, Claus Anders Bertelsen, Dario Baratti, Darren Au, David Clark, Declan Dunne, Denny Levett, Despoina Liotiri, Dimitrios Schizas, Dominique Engel, Dorian Krsul, Edoardo Virgilio, Ebrahim Ali Mohammed Amrein, Eleni Kitsou, Elharith Abduelfattah Gaber Mohammed, Elissavet Anestiadou, Emily Traer, Engy Elkoury, Enrique Alday Muñoz, Erik Nordenström, Erdinç Kamer, Eslam Elshenawy, Ewen Harrison, Fabio Ausania, Fadwa Fuad Abdulwahid Mansoor, Fatima Elmobark, Fernando Arduini, Fevzi Cengiz, Francesk Mulita, Francesco Mongelli, Francesco Pata, Frank Frizelle, Frederik Berrevoet, Gabrielle H. Van Ramshorst, Gianluca Pellino, Grainne Sheill, Gregg Nelson, Hadeer Alsadeg Mohammed, Hande Gurbuz, Hans D. De Boer, Hamid Aslami, Hamza Elhamzaoui, Hashem Abu Serhan, Haidara Bohsas, Heleen Driessens, Helena Pereira, Helmut Raab, Iacopo Cappellini, Ian Randall, Ianthe Boden, Ileana Lulic, Ilenia Merlini, Ina Ismiarti Shariffuddin, Jakub Chmelo, James Durrand, Janine Bothe, Jason Park, Jayson Moloney, Jenelle Loeliger, Jonathan Hardman, Joost Klaase, Jose Ignacio García-Sánchez, Julia Dubowitz, Julie Hallet, Junichiro Kawamura, Jyotirmay Kirtania, Kari Clifford, Kariem El-Boghdadly, Kate McBride, Khaled Abdelwahab, Khalid Osman Mohamed, Kheng-Seong Ng, Khloud Ali M. Dahan Saeed, Kimberley Bostock, Kilian Brown, Kristy-Lee Raso, Kyrillos Nassim, Kylie Hill, Kwong Weng Loh, Lara Edbrooke, Laura Lorenzon, Lewis Matthews, Linda A. Antonucci, Linda Edgar, Lissa Spencer, Louise Brennan, Magdalena Sielewicz, Magdalena Taube, Mahmoud Eissa, Majd Chaaban, Malcolm West, Malak Ghemmeid, Malek Balkhi, Manazir Athar, Marc Pocard, Marc Van De Velde, Marco Sparavigna, Marcos Gómez Ruiz, Marie-Louise Lydrup, María García Nebreda, Marta Ubre, Massimo Falconi, Michael Hii, Michael Kelly, Miguel Leon-Arellano, Moataz Maher Emara, Mohamed Al Gharyani, Mohamed Alsori, Mohamed Elbahnasawy, Mohamed Fouad Abdrabo, Mohamed Ghula, Mohamed Said, Mohamed Sherif Ali Ahmed, Mohammed Abdallah Mohammed Kheer Almotaared, Mohammed Al-Shehari, Mohammed Anass Majbar, Mohammed Izzaldin, Mohammed Omar, Mohammed Sarhan Alkhallatee, Mogahid Ahmed, Momin Hilles, Mostafa Algabri, Moyasar Awad, Msara Haider, Muhamad Kanjo, Nada Wd Badr, Nader Hanna, Nassila Krita, Natasa Kovac, Neil Pillinger, Nermina Rizvanovic, Nivedhyaa Srinivasaraghavan, Nurhilal Kiziltoprak, Orestis Ioannidis, Omar Ahmed Abdelwahab, Osman Osman, Oumayma Lahnaoui, Pankaj Roy, Pamela Klassen, Patrice Forget, Patricia Sylla, Paul Sutton, Pawel Kabata, Pawel Mroczkowski, Per J. Nilsson, Petra Bor, Peter Ihnát, Peter Noordzij, Peter Paal, Philip H. Pucher, Piotr Major, Qiang Wang, Rafat Muharam, Raquel García Álvarez, Rasha Shrayyef, Rashed Alweshah, Reham Atef Zaki Mohamed, Rhonda Farrell, Riccardo Caruso, Riyadh Ikreedeegh, Robert Arnold, Roberto Falz, Rohan Miegel, Rosalia Navarro-Perez, Ross Dolan, Safia Adem, Saleem Ahmed, Sami Sannoufa, Sandra Auld, Sara S. Elsheikh, Sarah Abdelmohsen, Sarah Braungart, Sasa Rajsic, Sawsan Ismail, Semra Demirli Atici, Seymanur Altintas Filizoglu, Shannon Philp, Sharon Carey, Shelby Yaceczko, Simone Manfredelli, Sorrel Burden, Sophie Hogan, Stefano Mancin, Styliani Apostolaki, Susana González-Suárez, Talat Abu Salem, Tania Triantafyllou, Tarik Sammour, Tim Bright, Tom Parkington, Varsha Gorey, Vasileios Mousafeiris, Varut Lohsiriwat, Victor Zaydfudim, Walter R. Frontera, Walid Ibrahim, Waled Aljbali, Wilton Van Klei, Yasemin Yıldırım, Yousef Abo Alsoud, Zakaria Belkhadir

**Affiliations:** 1 Surgical Outcomes Research Centre (SOuRCe), Royal Prince Alfred Hospital, Sydney, Australia; 2 Discipline of Physiotherapy, Graduate School of Health, Faculty of Health, University of Technology Sydney, Sydney, Australia; 3 Physiotherapy Department, Royal Prince Alfred Hospital, Sydney, Australia; 4 NHMRC Clinical Trials Centre, Faculty of Medicine and Health, The University of Sydney, Sydney, Australia; 5 Institute of Academic Surgery (IAS), Royal Prince Alfred Hospital, Sydney, Australia

## Past

Cancer surgery carries significant perioperative risk. Optimizing a patient’s health status before surgery, known as ‘prehabilitation,’ has emerged as a promising strategy to reduce postoperative complications and accelerate recovery. Despite growing evidence of its benefit, prehabilitation remains far from universally accessible. A recent global survey found that only 21% of hospitals provide prehabilitation as a standard of care,^1^ largely because of resource constraints and narrow timeframes between diagnosis and surgery. To address this, we set out to develop an online, self-reported screening tool through a comprehensive pre-defined process.^2^ Stage one involved a scoping review to identify screening tools used in patients undergoing gastrointestinal cancer surgery, yielding 77 unique tools across physical (n = 21), nutritional (n = 16), and psychological (n = 40) domains.^3^

## Present

In stage two, we conducted an international Delphi study^4^ to achieve expert consensus on which self-reported screening tools, identified through the scoping review, were appropriate for use. We recruited 308 multidisciplinary prehabilitation experts from 49 countries via the Global (P)rehabilitation Initiative (https://source.sydney.edu.au/global-prehabilitation-initiative/). This resulted in 10 self-reported screening tools that met consensus as appropriate across physical, nutritional, and psychological domains. Systematic evaluation of these tools, in terms of clinical utility and implementation feasibility, led to the identification of three optimal tools for use in the Preoperative Risk Evaluation for Cancer Treatment (PREdiCT) screening tool, to be tested in an international prospective cohort study (Stage 3).

## Future

The PREdiCT study will recruit 1214 patients from 36 sites across 19 countries, with the primary aim of establishing optimal risk stratification thresholds. If validated, PREdiCT has the potential to transform how we identify and support vulnerable patients during their cancer experience. By enabling clinicians to rapidly and accurately identify people at high risk, the tool will facilitate the delivery of targeted and tailored prehabilitation, ensuring that resources are directed where they are most needed.
